# Realizing Crack Diagnosing and Self‐Healing by Electricity with a Dynamic Crosslinked Flexible Polyurethane Composite

**DOI:** 10.1002/advs.201800101

**Published:** 2018-03-08

**Authors:** Wuli Pu, Daihua Fu, Zhanhua Wang, Xinpeng Gan, Xili Lu, Li Yang, Hesheng Xia

**Affiliations:** ^1^ State Key Laboratory of Polymer Materials Engineering Polymer Research Institute Sichuan University Chengdu 610065 China

**Keywords:** carbon nanotubes, crack diagnosing, polyurethane, self‐healing

## Abstract

Combining self‐healing functions with damage diagnosing, which can achieve timely healing autonomously, is expected to improve the reliability and reduce life cycle cost of materials. Here, a flexible conductive composite composed of a dynamically crosslinked polyurethane bearing Diels–Alder bonds (PUDA) and carbon nanotubes (CNTs), which possess both crack diagnosing and self‐healing functions, is reported. The introduced dynamic Diels–Alder bonds endow the materials self‐healing function and the powder‐based preparation route based on the specially designed CNTs‐coated PUDA micropowders leads to the formation of segregated CNTs network, which makes the composite possess excellent mechanical properties and high conductivity. Because of the sufficient electrothermal and photothermal effect of CNTs, the composites can be healed rapidly and repeatedly by electricity or near‐infrared light based on the retro‐Diels–Alder reaction. An obvious color difference in the infrared thermograph resulting from the resistance difference between damaged and undamaged area can be observed when applying the voltage, which can be used for crack diagnosing. Using the same electrical circuit, the crack in the PUDA/CNTs composite can be noninvasively detected first and then be autonomously healed. The composites also exhibit a strain‐sensing function with good sensitivity and high reliability, thus will have potential applications in electronic strain sensors.

Polymer materials are susceptible to be damaged, which will severely weaken the mechanical performance and lifetime of materials. Early damage diagnosing and self‐healing is highly required for preventing the catastrophic failure and reducing the economic losses. For early damage diagnosing, an extensive strategy is embedding microcapsules or hollow fibers containing visual indicators such as fluorescence agents,^1^, ^2^, ^3^ pH‐sensitive dyes,^4^ and luminogen^5^, ^6^ into polymer matrix. Upon mechanical damage, the microcapsules will rupture and release the visual indicators to generate a signal to detect.^7^, ^8^ Using mechanochemically responsive polymer containing mechanophores which can convert mechanical stimuli into an optical response displaying color and/or fluorescence changes was also proposed.^9^, ^10^, ^11^, ^12^, ^13^, ^14^, ^15^ Those damage‐reporting strategies can be generally utilized to detect the cracks on the surface where the color or fluorescence change is visual, while the cracks formed inside the bulk materials are often difficult to be detected, and thus it is necessary to develop more effective crack diagnosing method.

The intrinsic self‐healing materials, which can achieve multiple repair cycles through inherent reversible chemical bonds, have attracted increasing attention. Reversible systems based on covalent interactions, such as Diels––Alder,^16^, ^17^, ^18^, ^19^ chain reshuffling reaction,^20^, ^21^, ^22^ and noncovalent interactions, such as hydrogen bonding,^23^, ^24^, ^25^ metal–ligand coordination,^26^, ^27^, ^28^ host–guest interactions,^29^ π–π stacking,^30^ or electrostatic interaction,^31^, ^32^ were well studied. The healing process normally needs external stimulus (e.g., heat,^16^ light,^33^, ^34^ redox,^29^ ultrasound,^35^, ^36^ and electricity^37^) to trigger the reversible reaction. Compared to other stimulus, light as a kind of economical energy can be remotely controlled and the light intensity can be focused precisely onto the damaged areas without interference of other parts. Near‐infrared (NIR) light‐responsive self‐healing materials with carbon nanotubes (CNTs)^38^ and graphene^39^ as fillers have been investigated. Electricity can be input locally and thus is a good stimulus approach to trigger the healing process for electrical conductive materials. Raquez and co‐workers fabricated thermoreversible and electrically conductive poly(ester‐urethane)/CNTs composites, which can be locally healed under electrical current.^40^ In order to adapt to different situations, the multiple stimuli used for triggering the self‐healing process are required.^41^ Furthermore, combining self‐healing function with damage diagnosing which can achieve timely healing autonomously is highly expected for improving reliability and reducing life cycle cost of materials; however, the related research work is rarely reported.

In recent years, introducing self‐healing properties to conductive materials which can effectively enhance the reliability, durability, and functionality of the electronic device has been intensively investigated.^42^, ^43^ A range of electronic devices with self‐healing functionality, such as supercapacitor,^44^ electronic skin,^45^ electronic sensors,^46^ conductor,^47^, ^48^ and lithium ion batteries^49^ were developed. The conductive self‐healing materials are fabricated by spreading inorganic silver nanowires,^50^ CNTs^51^ onto the surface of self‐healing polymer substrates or embedding conductive microparticles,^52^ such as liquid metal or ionic liquids^48^, ^53^ inside polymer matrix. Recently, some conductive self‐healing flexible elastomers filled with CNTs, constructed by host–guest interactions or supramolecular multiple hydrogen bonding, were developed and used as reliable sensors.^54^, ^55^ Their high flexibility and excellent ambient temperature self‐healing ability was demonstrated; however, the mechanical properties are relative low and the preparation processes are complicated. Therefore, the development of flexible self‐healing materials with highly conductivity and good mechanical properties is desirable for electronic devices.

In order to overcome the issues mentioned above, herein, we realize the electricity managed crack diagnosing and self‐healing functions based on the highly conductive dynamic crosslinked polyurethane/CNTs composites prepared through a powder‐based processing route, that is, hot compression of the CNTs‐wrapped polyurethane bearing Diels–Alder bonds (PUDA) powders. The dynamic Diels‐Alder (DA) bonds are introduced into the polyurethane chain by molecular engineering to endow the materials self‐healing function. Using the powder‐based processing route, not only good mechanical properties can be obtained, but also the electrical conductive segregated network of CNTs can be constructed in the PUDA matrix, which dramatically enhances the electrical conductivity and reduces the percolation threshold. The excellent electrical conductive properties endow the materials damage‐reporting and electricity triggered self‐healing functions. When mechanical damage occurs, the resistance in crack site is higher than that in other intact parts, resulting in an intensive electrothermal effect and color difference in the infrared (IR) image under applied voltage, which provides an excellent method for crack diagnosing. Under prolonged electricity, the sufficient heat will be produced to further initiate the retro‐DA reaction to heal the crack. In addition, the healing of PUDA/CNTs composites can be accomplished by subjecting the damaged areas to a low‐power NIR light irradiation owing to the strong NIR absorption of CNTs. The PUDA/CNTs composites also show strain‐sensing capability and thus display promising applications as the electronic sensor.

A hierarchical structure design strategy of PUDA/CNTs composite: from the molecular engineering to CNTs segregation network construction, was adopted, as schematically illustrated in **Figure** **1** (also see the Experimental Section for details). First, the dynamically crosslinked PUDA bearing Diels–Alder bonds was prepared according to our previous work^36^ as shown in Scheme S1 (Supporting Information). The dynamic DA bonds are distributed in the main chain and crosslink points of polyurethane molecules by molecular design to ensure a good healing property. The prepared bulk PUDA was then cryogenically pulverized and sieved to obtain the PUDA powders with an average size of ≈50 micrometers. Then CNTs wrapped PUDA powders were prepared through a liquid phase adsorption and deposition process according to the following procedure. The CNTs are uniformly dispersed into the ethanol by ultrasonication method. After the addition of PUDA powders in the CNTs ethanol dispersion, the CNTs were rapidly adsorbed onto the surface of the PUDA powders due to the electrostatic adsorption effect between PUDA and CNTs, resulting in the precipitation of the mixture to obtain PUDA/CNTs composite powders (Figure S1, Supporting Information). The PUDA powders before and after wrapped with CNTs were characterized using scanning electron microscopy (SEM), as shown in **Figure** **2**a,b and Figure S2 (Supporting Information). After wrapping by CNTs, the surface of the PUDA powder becomes flocky due to the existence of the 1D CNTs. There is no particles adhesion between CNTs wrapped PUDA powders which is the same as the uncoated one. The particle‐size distribution of the PUDA/CNTs composite powders with 1 wt% CNTs loading is shown in Figure 2c. The PUDA/CNTs composite powders have an average size of ≈50 micrometers, nearly the same as the uncoated PUDA powders, indicating that the coated CNTs layer is not so thick. Based on the self‐made CNTs‐coated PUDA micropowders, the highly conductive PUDA/CNTs composites were fabricated through a simple compression molding process.

**Figure 1:**
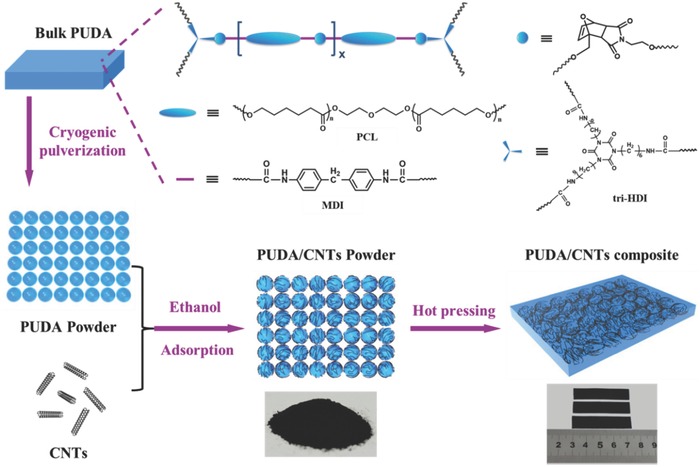
Schematic illustration of the hierarchical structure design strategy of PUDA/CNTs composite: from the molecular engineering to CNTs segregation network construction.

**Figure 2:**
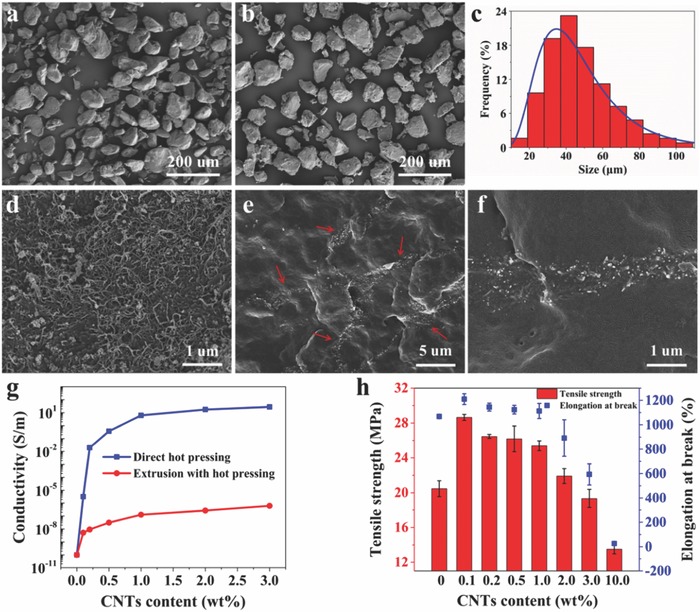
SEM images of a) neat PUDA powders obtained through cryogenic pulverizing and b) CNTs wrapped PUDA composite powders with 1 wt% CNTs loading. c) Particle‐size distribution histogram of CNTs wrapped PUDA composite powders with 1 wt% CNTs loading. d) SEM image of the surface morphology of CNTs wrapped PUDA composite powders with 1 wt% CNTs content. e) Low and f) high magnification cross‐sectional SEM images of compression molded PUDA/CNTs composite prepared using CNTs wrapped PUDA composite powders with 1 wt% CNTs content. g) Electrical conductivities of PUDA/CNTs composites with different CNTs contents prepared via two different methods. h) Tensile properties of neat PUDA and PUDA/CNTs composites with different CNTs contents.

The dispersion of 1 wt% CNTs loading on the surface of PUDA powders is shown in Figure 2d. No obvious aggregation of CNTs was observed, implying good dispersion of CNTs in the surface and strong interaction between PUDA and CNTs. PUDA/CNTs powders with different CNTs contents were prepared, and it is obvious that more CNTs could be coated on the surface of PUDA powders with the increase of CNTs contents (Figure S3, Supporting Information). Figure 2e,f and Figure S4 (Supporting Information) show the cross‐section morphology of the PUDA/CNTs composites with different CNTs contents. As the compression molding is a static processing without shearing like injection molding, the wrapped CNTs can form segregation networks in the PUDA matrix through the powder‐based processing route, which will dramatically enhance the electrical conductivity. The segregated network starts to form with the addition of CNTs at ≈0.2 wt% (Figure S4, Supporting Information), and more complete CNTs networks were obtained with the increase of CNTs content. Figure 2e,f shows the CNTs segregation networks at a CNTs content of 1 wt%. The electrical conductivities of PUDA/CNTs composites were measured using a two‐probe technique, and the variation with CNTs contents is shown in Figure 2g. The conductivity shows an abrupt increase upon adding 0.2 wt% CNTs, implying the occurrence of electrical percolation. At a CNTs content of 3.0 wt%, the electrical conductivity of the composite could reach as high as 27.9 S m^−1^. According to the classical percolation theory, the relation between electrical conductivity, σ, of a polymer/filler composite material and filler concentration can be described via a simple power law as shown in Equation (1)

(1) $$\sigma \propto {\left(x-x_{c}\right)}^{\alpha }$$

where *x* is the volume fraction of the filler, *x*_c_ is the volume fraction of the percolation threshold, and α is the critical exponent which is dependent on the system dimension. The fitting to the experimental values results in *x*_c_ of 0.0005 and α of 1.41, indicating a very low percolation threshold in PUDA/CNTs system. The critical exponent α by theoretical prediction ranges from 1.2 to 1.6 means that the CNTs network is a 3D percolating system.^56^ In comparison, the electrical conductivities of PUDA/CNTs composites prepared through extrusion combined with hot pressing are much lower than the composites from direct hot pressing without extrusion (Figure 2g), for example, at a CNTs content of 1 wt%, the electrical conductivity for the latter one is approximately eight orders of magnitude than that of the former one. This is because that the shearing process during the extrusion can break the structure of CNTs networks.^57^, ^58^ This observation demonstrated that our proposed powder‐based compression molding approach is very effective to contribute to a significant increase in the conductivity of PUDA/CNTs composites.

The mechanical properties of PUDA and PUDA/CNTs composites were investigated. Figure 2h shows that the maximum tensile stress and elongation at break can be enhanced with CNTs addition. Detailed mechanical properties are shown in Table S1 (Supporting Information). At 1% CNTs content, the tensile strength and elongation at break of the PUDA/CNTs composite are ≈25.8 MPa and ≈1052%, respectively, much higher than the reported flexible conductive elastomer.^54^, ^55^ The enhanced mechanical strength is owing to the outstanding mechanical property of CNTs and the formation of CNTs networks in the matrix. The optimized mechanical properties appears at 0.1 wt% CNTs content and when the CNTs content is over 3.0 wt%, the mechanical properties show a decreasing trend. When the CNTs content is up to 10.0 wt%, the composite becomes very fragile due to the increased physical crosslink of CNTs.^59^

The conductive PUDA/CNTs composites show outstanding crack diagnosing and self‐healing capabilities under electricity stimulus. The electricity‐triggered crack diagnosing and self‐healing concept of PUDA/CNTs composites can be illustrated in **Figure** **3**a. Owing to the high electrical conductivity, the PUDA/CNTs composites possess remarkable electrothermal capability of efficiently transducing electrical energy into Joule heating energy, resulting in a fast heating rate and a high equilibrium temperature at a relatively low applied voltage (less than 40 V) (Figures S7 and S8, Supporting Information). When the crack is formed, a slight resistance increase of PUDA/CNTs composites will appear (Figures S9 and S10, Supporting Information). However, even under severe damage that the composite was cut completely with a little joint left, the resistance is only twice the original value (Table S2, Supporting Information), implying that the PUDA/CNTs composites can maintain their good electrical conductivity once damaged. Moreover, upon the formation of crack, a larger electrothermal effect is generated at the damaged area as the resistance in the fractured area is higher than that in other parts of the composites.^41^, ^60^ As a result, when damaged, the PUDA/CNTs composites can show color difference under an Infrared Thermal Imager between the damaged area and other intact parts after applying a voltage, and thus can indicate the location of crack. Meanwhile, the reversible retro‐DA reaction can occur in damage area at higher temperature and consequently the PUDA network reorganization occurs, which leads to the self‐healing behaviors of PUDA/CNTs composites.

**Figure 3:**
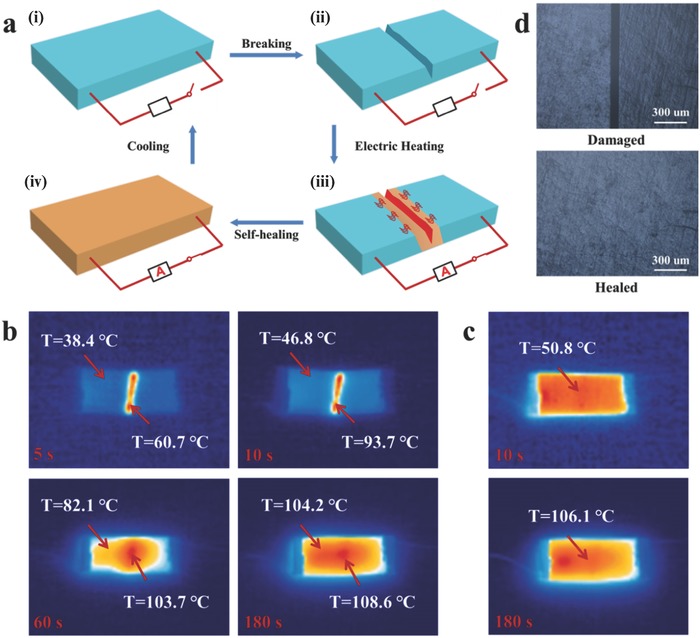
a) Schematic representation of autonomic electricity‐triggered damage reporting and self‐healing concept of PUDA/CNTs composite as an electronic device: (i) undamaged composite; (ii) cut the composite to form a crack; (iii) applying an appropriate voltage; and (iv) completely healed. b) The surface temperature of PUDA/CNTs composite with 1 wt% CNTs content recorded by an Infrared Thermal Imager at different stages during the electricity‐triggered damage reporting and healing process. c) The surface temperature of the healed composite subject to a second electricity heating. The applied voltage was 20 V. d) Optical microscope images of damaged sample and healed crack for PUDA/CNTs composite with 1 wt% CNTs content.

To investigate the electricity‐triggered damage detection and healing process, the PUDA/CNTs sample (25 mm × 10 mm × 0.85 mm) was cut in the middle to form a crack along the entire length in a direction perpendicular to the current flow. The surface temperature of the PUDA/CNTs composite at 1 wt% CNTs content during electricity heating process at a voltage of 20 V is recorded with an Infrared Thermal Imager (Figure 3b). It should be pointed out that the recorded thermal images were intentionally set in the opposite surface to crack side for a purpose to illustrate the internal damage detection function. Once a voltage of 20 V is applied, the temperature in the damaged area quickly increases to 60.7 °C in 5 s, which is much higher than the temperature of ≈38.4 °C at the same time in other intact parts. The temperature in the crack area can reach 93.7 °C in 10 s, while any 46.8 °C in the other intact parts. Clearly, the electricity‐induced heating rate for the PUDA/CNTs composite in the damaged area is much higher than that in the intact areas due to a higher electrical resistance. After 180 s, the color of IR thermal image for the damaged area closes to that for the other intact parts, indicating that the damage was healed. The excellent self‐healing performance was further verified by recording the second electricity heating process of the healed sample (Figure 3c). The healed sample shows uniform surface temperature in the healed crack site and other intact parts, demonstrating the disappearance of the crack after complete healing. From the optical microscope images shown in Figure 3d, we can clearly see that the obvious fracture of the cut‐off sample vanishes completely after healing process. The healing behavior also can be demonstrated from the appearance pictures and 3D optical images (Figure S11, Supporting Information). Three “damage‐healing” cycles were conducted and the results demonstrated excellent repeatable crack diagnosing and self‐healing functions in the PUDA/CNTs conductive composite (Figure S12, Supporting Information). Under the DC electricity, the PUDA/CNTs composites can heal themselves autonomously once the cracks are formed as the cracks can response to the voltage through electrothermal effect and show high temperature to induce healing behavior. Thus, PUDA/CNTs composites integrated the damage reporting and autonomously healing function by electricity will possess promising applications as electronic devices and composites.

The PUDA/CNTs composite can recover its mechanical properties after self‐healing via electricity (**Figure** **4**a and Figure S13, Supporting Information). To investigate the mechanical self‐healing performance during different healing stages, we define the healing efficiencies as the ratio of the tensile strength of the healed sample to the original sample. Figure 4a shows the representative stress–strain curves for the original, damaged, and healed PUDA/CNTs composite with 1 wt% CNTs content at different treatment time under electricity. The damaged sample after cutting has an elongation of only ≈38% compared to ≈1178% of the original one and also shows a very low tensile strength. Once the sample was applied a voltage of 30 V, the strain and stress increase with time. After 180 s, the sample exhibits almost the same mechanical properties as the original and the healing efficiency can reach ≈98%, showing a nearly complete healing. The rapid healing process can be attributed to the faster heating rate in damaged area. Under three “damage‐healing” cycles, the healing efficiency for the first, second, and third“damage‐healing” cycles is ≈98%, ≈93%, and ≈83%, respectively, suggesting that the materials can be healed for multiple times with a slight decrease in the healing efficiency (Figure S14a, Supporting Information). The applied voltage shows a significant effect on the mechanical healing ability of PUDA/CNTs. Normally the healing efficiency increases with increasing the voltage, but a long time heating at high voltages may degrade the materials, leading to a low healing efficiency (Figure S14b, Supporting Information). The PUDA/CNTs with high CNTs loading can obtain their best healing efficiencies under a relative low voltage. However, too many CNTs can hinder the retro‐DA reaction and restrict the polymer chain mobility (Figure S15, Supporting Information). It should be pointed out that the PUDA/CNTs samples with a CNTs content of lower than 0.5 wt% cannot be healed through electricity because of their low electrical conductivity and insufficient electrothermal effect.

**Figure 4:**
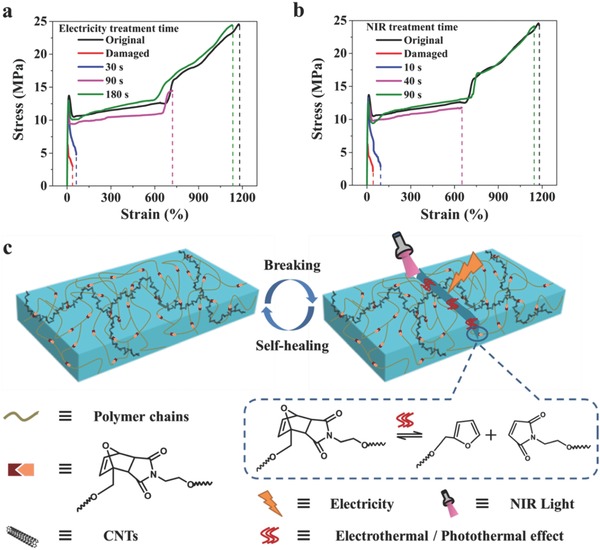
a) Stress–strain behavior of damaged and healed PUDA/CNTs composite with 1 wt% CNTs content under different electricity treatment time (applied voltage: 30 V). b) Stress–strain behavior of damaged and healed PUDA/CNTs composite with 1 wt% CNTs content under different NIR light treatment time (NIR light intensity: 0.4 W cm^−2^). c) Schematic illustration of self‐healing process of PUDA/CNTs composite triggered via electricity and NIR light stimulus.

Alternatively, the self‐healing of the PUDA/CNTs composites can be achieved by subjecting the damaged area to a low power NIR light irradiation owing to the efficient NIR absorption and photothermal capacity of CNTs (Figures S16 and S17, Supporting Information). As shown in Figure 4b, the healed PUDA/CNTs composite with 1 wt% CNTs content almost completely recovers its original tensile stress and elongation at break after only 90 s NIR light exposure with an intensity of 0.4 W cm^−2^, showing a rapid healing efficiency of ≈97% and excellent repeatable healing ability (Figure S18a, Supporting Information). A higher healing efficiency can be achieved under a higher NIR light intensity and a longer exposure time (Figure S18b, Supporting Information). The NIR light can focus its energy on a spot area and thus can localize the heating only in a selected part of the polymer without affecting other place, showing its significant advantage compared to other healing methods.

The self‐healing mechanism of the conductive PUDA/CNTs composites through electricity and NIR light irradiation is schematically illustrated in Figure 4c. The thermal‐induced self‐healing mechanism of reversible covalent crosslinking polymer materials based on the DA bonds has been investigated.^16^ The furan–maleimide adducts in the PUDA materials, as a promising motif, can reversibly undergo retro‐DA reaction and DA reaction at a higher temperature induced by electrothermal or photothermal effect of CNTs, which causes a concomitant reversible degradation of the polymer network, followed by wetting, diffusion, and entanglement of the temporary lower‐molecular‐mass species in two crack surfaces. The dissociated furan and maleimide moieties may experience rebonding or reshuffling reactions to form cycloaddition adducts upon cooling, leading to the self‐healing. In our experimental setup, the remolding behavior by hot pressing used to prepare the composite from the PUDA/CNTs powders suggested the thermal‐induced reconstruction of crosslinked PUDA network can happen.

To further investigate the crack diagnosing and self‐healing performance of the PUDA/CNTs composite in more situations, we cut the PUDA/CNTs composite sample (25 mm × 10 mm × 0.85 mm) to form more cracks in different locations. As shown in **Figure** **5**a (i), once applied an appropriate voltage, the temperature at the crack area was much higher than other parts and the PUDA/CNTs composite showed different colors in IR thermal images in 10 s. The red color as a sign of higher temperature means the location of the crack, demonstrating the damage‐reporting ability of PUDA/CNTs composites when used as electronic devices. No matter where the location of the crack is, the color difference at the crack site can be exhibited in IR thermal images at the early stage. Even though there are many cracks, the color difference at every crack was highly visible in comparison to other intact parts. More importantly, the surface temperature shown in IR thermal images was obtained by measuring the opposite side of the cracks, which suggests that the crack of PUDA/CNTs composites can be indicated from the reverse side, showing a noncontacting and noninvasive detection capability. After applying a 20 V voltage for 180 s, as shown in Figure 5a (ii), all the color distinction between damaged area and intact parts disappears, providing a secondary indication that all the damages were healed.

**Figure 5:**
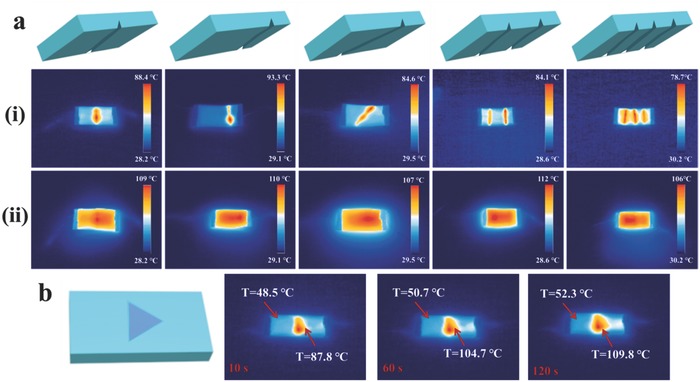
a) The surface temperature of PUDA/CNTs (1 wt%) composite sample with different crack types and numbers recorded by an Infrared Thermal Imager under an applied voltage of 20 V (i) for 10 s and (ii) for 180 s, demonstrating the autonomous damage indication in IR images and self‐healing ability of PUDA/CNTs composite induced by electricity, respectively. b) The surface temperature change of the PUDA/CNTs composite embedded an insulated triangle with 0.4 mm thickness under an applied voltage for different time.

In order to better illustrate the crack diagnosing function inside the composites, we put an insulated triangle paper with 0.4 mm thickness inside the PUDA/CNTs composite matrix and then recorded the IR thermal images under voltage with different time. As shown in Figure 5b, the area where we put the insulated object shows red color obviously and illustrates a shape of a similar triangle due to the higher temperature than other parts in 10 s. After applied voltage for 120 s, the composite still remains the red color as the self‐healing process at the damage cannot occur in this case. This demonstrated that the invisible crack inside the conductive PUDA/CNTs composites can be detected noninvasively through electricity which is difficult to be achieved via other approaches.

The healing in the electrical conductivity properties is equally important as mechanical strength to ensure their reliability. To investigate NIR‐triggered electrical conductivity recovery property, the PUDA/CNTs sample with 1 wt% CNTs content (45 mm × 10 mm × 0.85 mm) was completely cut into two parts from the middle and the two fractured surfaces were brought together along with NIR light irradiation. The resistance variation of the PUDA/CNTs composite is apparently shown in **Figure** **6**a–d and the resistance increases slightly by ≈10% after self‐healing. Figure 6f describes the time‐dependent resistance variation in a typical electrical conductivity recovery process from damage to be healed. Once the sample is completely bifurcated, the resistance of the PUDA/CNTs composite increases from ≈670 Ω to infinite as the formation of an open circuit. When the two fractured edges of the composite are brought into contact, the resistance rapidly drops to ≈10^6^ Ω due to the contact of the conducting CNTs layer on the surface, but also showing very low conductivity. After the sample is treated by NIR light irradiation (0.4 W cm^−2^), the sample resistance further decreases and returns to its initial value within 20 s, suggesting an excellent electrical healing performance. It can be attributed to the reorganization of polymer chains and the reconnection of CNTs networks induced by the retro‐DA reaction under NIR light irradiation. Figure 6g demonstrates that the PUDA/CNTs composites exhibit excellent repeatable recovery of electrical conductivity after undergoing five times cuts at the same damaged location.

**Figure 6:**
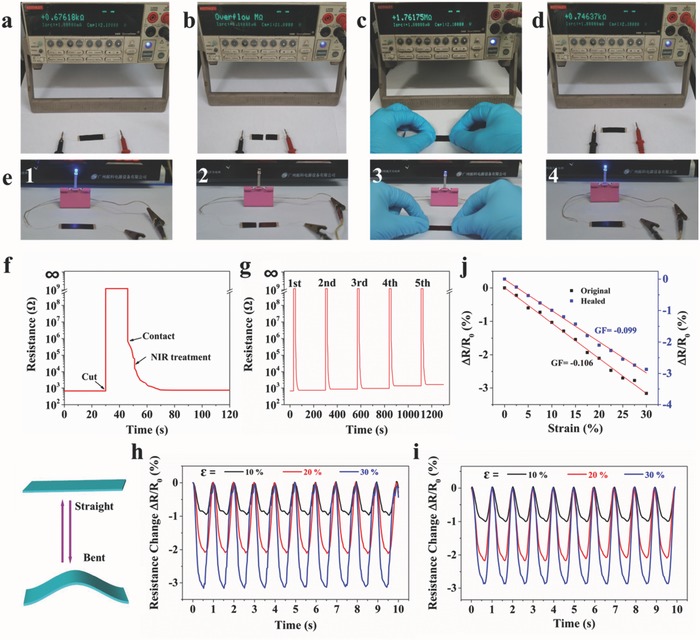
a–d) Electrical resistance of the PUDA/CNTs composite sample in a) original, b) bifurcated, c) contacted, and d) healed stage. e) Photographs of PUDA/CNTs composite sample as a self‐healing electrical conductor to power an LED lamp during healing process. 1, undamaged conductor; 2, completely breaking; 3, contacting; and 4, electrical healing by NIR light irradiation (0.4 W cm^−2^) method. f) Electrical resistance variation of the PUDA/CNTs composite sample during healing process using NIR light irradiation (0.4 W cm^−2^) method. g) Repeated electrical conductivity recovery performance in five damaged‐healing cycles at the same fractured location. h,i) Resistance change (▵*R*/*R*_0_) versus time under different applied strain in compressive cyclic bending modes of h) original PUDA/CNTs composite and i) self‐healed PUDA/CNTs composite. j) Resistance change (▵*R*/*R*_0_) versus applied strain of original and self‐healed PUDA/CNTs composite. (The CNTs content was 1 wt%.)

Furthermore, we investigated the electrical performance recovery of the PUDA/CNTs composite when used as electronic circuits. Figure 6e shows the healing process of the PUDA/CNTs composite sample as a conductor to power a light‐emitting diode (LED) lamp in a circuit. The LED lamp could successfully light up at an applied voltage of 10 V. Upon completely breaking the sample into half, the LED turned off as the circuit fractured. The LED lamp was lit up again and showed a weak brightness when the two cross‐sections contacted. After completely self‐healing using NIR light irradiation method, the LED lamp was able to recover its original brightness. The brightness variation of the composite is corresponding to the resistance change during healing process.

To investigate the strain‐sensing performance of the composites, we measured the time‐dependent resistance changes during repetitive mechanical bending deformation for the PUDA/CNTs sample with 1 wt% CNTs content. Figure 6h,i shows the normalized resistance ▵*R*/*R*_0_ versus time curves under various strains in the bending strain test (one cycle per second) of the original sample and self‐healed sample, respectively. For the original sample, the 10% bending strain leads to a decrease of ≈1% in the resistance due to the decrease in the gaps between the conductive networks. When the applied bending strain increases from 10% to 30%, the resistance change becomes more obvious. This bending–straightening process can be repeated many times without any apparent loss of the resistance change. Notably, the self‐healed sample also shows the same strain‐sensing capability which is highly consistent with original sample. We further evaluated the stability in strain sensing of the PUDA/CNTs composite with 1 wt% CNTs. Figures S20 and S21 (Supporting Information) show the resistance variation of the original sample and self‐healed sample during cyclic bending–straightening tests (1000 cycles) at a frequency of three or five cycles per second at a strain of 20%. Both the original sample and self‐healed sample maintain similar sensitivity after bending over 1000 cycles even at a high bending frequency. There is no obvious increase in the resistance of the original sample and the healed sample after 5000 cycles of bending–straightening test (Figure S22, Supporting Information), showing good stability as a strain sensor. To evaluate the sensitivity of the composite to various applied strains, gauge factor (GF) which is defined as the ratio of relative resistance change (▵*R*/*R*_0_) versus applied strain is calculated. As shown in Figure 6j, the original sample shows a GF of −0.106 when applied less than 30% strain, while the self‐healed sample exhibits a slightly lower GF of −0.099. The results demonstrate that the self‐healing PUDA/CNTs composite possesses relative good sensitivity, high durability, and reliability in strain‐sensing process, and thus have promising potential application in electronic devices.

In summary, we have demonstrated a kind of flexible PUDA/CNTs composite with crack diagnosing, self‐healing, as well as excellent mechanical properties. A hierarchical structure design strategy of PUDA/CNTs composite including molecular engineering of the dynamic DA bond for self‐healing and CNTs segregation network construction for electrical conductive property using the self‐made CNTs‐coated PUDA powders was adopted. When applied voltage, these composites show outstanding damage detection ability through the temperature difference in the IR thermal image, resulting from different resistance between the damaged area and the other intact parts. The composite can be heated once applied voltage or low‐power NIR light irradiation due to the excellent electrothermal and photothermal conversion capabilities induced by the addition of CNTs. Because of the dynamic DA chemical reaction at higher temperature, the PUDA/CNTs composites can be healed effectively and rapidly by electricity or NIR light irradiation in a very short time. The PUDA/CNTs materials with crack diagnosing and self‐healing functions exhibit good strain response capability, thus have great potential applications in electronic sensors.

## Experimental Section

*Materials*: Polycaprolactone diol (PCL, *M*_n_≈4000 g mol^−1^, Pershorp, Capa2402) was dried under vacuum at 110 °C for 12 h prior to use. 4, 4′‐Diphenylmethane diisocyanate (MDI, *M*_n_ = 250 g mol^−1^) was purchased from Yantai Wanhua Group. Hexamethylene diisocyanate trimer (tri‐HDI) was used as crosslinking agent without further purification. The 1‐(hydroxymethyl)‐10‐oxatricyclo[5.2.1.0^2,6^] dec‐8‐ene‐3,5‐dione‐2‐aminoethanol compound with Diels–Alder bond was synthesized by the same synthetic routes as Lu.^36^ Anhydrous 1, 4‐dioxane was obtained by distillation over sodium benzophenone. CNTs (NC7000) were obtained from Nanocyl, Belgium, which has an average diameter of 10–15 nm and length of 1.5 µm.

*Synthesis of Self‐Healing PUDA*: The dynamic crosslinked (20% crosslinking degree) self‐healing PUDA was synthesized following the synthetic routes as shown in Scheme S1 (Supporting Information). First, the NCO terminated PCL prepolymer was synthesized by the reaction of MDI and PCL with a molar ratio of 2:1 at 80 °C under nitrogen atmosphere for 2 h. Then the compound with DA bond, the prepolymer, and tri‐HDI was dissolved in anhydrous 1, 4 – dioxane with a molar ratio of 60:48:8. The mixture was degassed under reduced pressure and then cast into Teflon molds. Polymerization reaction and evaporation of the solvent took place at 80 °C under nitrogen atmosphere for 96 h. The polymer was further dried under vacuum to obtain a pale yellow nontransparent solid.

*Fabrication of PUDA/CNTs Composites*: The bulk PUDA material prepared through the above process was pulverized to get the PUDA powders using liquid nitrogen cryogenic pulverizing machine (250, Jiangyin Zhongkai Machinery Factory, China) at a temperature of −100 °C. Then the PUDA powders were sieved to remove big particles. To prepare the PUDA/CNTs composite powders, CNTs (0.1–3 wt%) were dispersed in ethanol by ultrasonication for 1 h. Then, the PUDA powders were added into the suspension with mechanical stirring. After stirring for 30 min, the suspension was filtered with a Buchner funnel under reduced pressure. The obtained particles were dried in a vacuum oven at 35 °C for 24 h. The PUDA/CNTs composite powders with different CNTs content were then hot pressed using a hot press machine (R‐32022015, Wuhan Qis'en Science and Technology Development Co., Ltd, China) under 9 MPa pressure at 100 °C to obtain the PUDA/CNTs composite samples.

*Characterizations of Materials*: The surface morphology of CNTs/PUDA powders and the dispersion of CNTs in PUDA matrix were observed on an FEI‐Quanta 250 field emission scanning electron microscope (SEM) at an acceleration voltage of 20 kV. Differential scanning calorimetry (DSC) was performed by using the TA Instrument Q20 DSC thermoanalyzer under nitrogen atmosphere at a heating rate of 5 °C min^−1^. Dynamic mechanical thermal analysis experiments were measured by a TA Instrument Q800 machine in the dual cantilever mode. The test temperature was from −80 to 100 °C and the heating rate was 3 °C min^−1^. The strain amplitude used was 50 µm and the stretching frequency was 1 Hz. Mechanical tensile–stress experiments were conducted on an Instron 5567 machine (USA) at room temperature with a strain rate of 50 mm min^−1^. The dimension of specimens is 45 mm × 10 mm × 0.85 mm. At least four samples of each loading fraction were tested. The electrical resistance was measured by two‐point probe resistance measurement system using a Keithley 2400 source meter (USA). The silver paste was coated on two sides to use as the electrode. The fracture surface was observed by an optical microscope (VHX‐1000, KEYENCE, Japan). The depth of cracks was measured by using laser scanning confocal microscope (LSCM). The samples for the test were added using Rhodamine B in the preparation process to show the fluorescent under LSCM. Infrared thermal images and the temperature variation under applied voltage or NIR light irradiation were recorded by a Testo 875i Infrared Thermal Imager. The bending–straight test was implemented by using Materials Test System, and the resistance signals of PUDA/CNTs composite were measured in real‐time using a Keithley 2510 source meter (USA).

*Self‐Healing Test*: Before healing test, a sheet sample of PUDA/CNTs composites (45 mm × 10 mm × 0.85 mm) was cut to form an 8 mm crack in the middle along the traverse direction. For calculating the mechanical healing efficiencies, at least three samples of each healing condition were tested. The NIR light‐triggered healing process was carried out by 808 nm Infrared Laser (LLR808nm 5W, China). The electricity‐triggered healing process was carried out by an Adjustable Voltage Switching Power Supply (YK‐AD15010, China).

## Conflict of Interest

The authors declare no conflict of interest.

## Supporting information

Supplementary
