# Supplementary material for: Realizing Crack Diagnosing and Self‐Healing by Electricity with a Dynamic Crosslinked Flexible Polyurethane Composite
Source: Adv Sci (Weinh). 2018 Mar 8;5(5):1800101. doi: 10.1002/advs.201800101 (PMC5978978; doi:10.1002/advs.201800101)
Supplement: Supplementary file 1 — Supplementary [file ADVS-5-1800101-s001.pdf]

Copyright WILEY-VCH Verlag GmbH & Co. KGaA, 69469 Weinheim, Germany, 2018.

ADVANCED SCIENCE
Open Access

Supporting Information

for *Adv. Sci.,* DOI: 10.1002/advs.201800101

Realizing Crack Diagnosing and Self-Healing by Electricity with a Dynamic Crosslinked Flexible Polyurethane Composite

*Wuli Pu, Daihua Fu, Zhanhua Wang, Xinpeng Gan, Xili Lu, Li Yang, and Hesheng Xia**

Copyright WILEY-VCH Verlag GmbH & Co. KGaA, 69469 Weinheim, Germany, 2018.

Supporting Information

Realizing Crack Diagnosing and Self-Healing by Electricity with a Dynamic Crosslinked Flexible Polyurethane Composite

*Wuli Pu, Daihua Fu, Zhanhua Wang, Xinpeng Gan, Xili Lu, Li Yang, Hesheng Xia**

E-mail: xiahs@scu.edu.cn

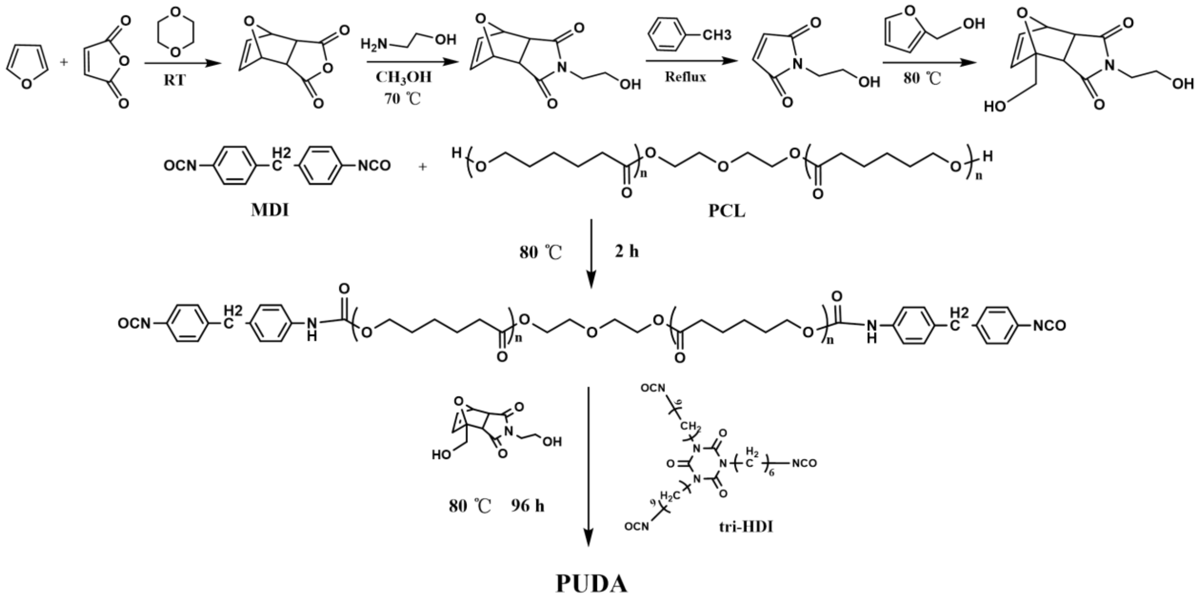

Scheme S1. Synthesis routes to the covalently cross-linked dynamic polymer PUDA based on DA bonds.

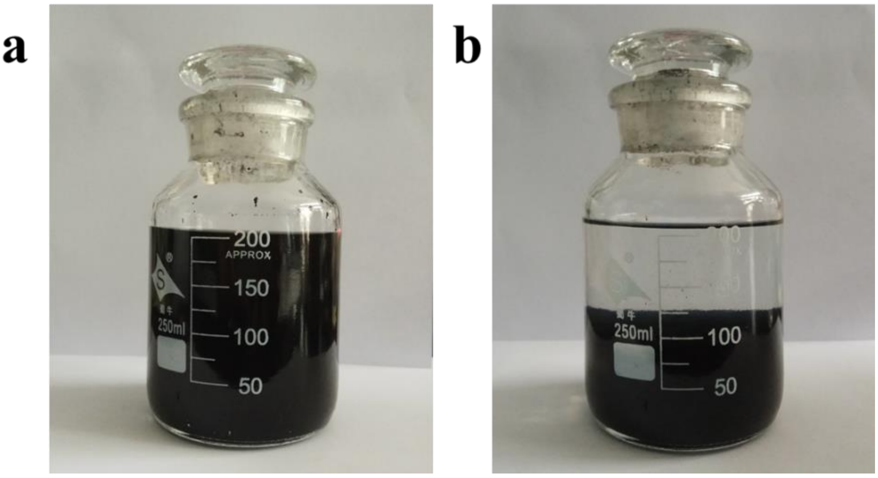

Figure S1. Photographs of (a) the mixture of CNTs dispersed in ethanol and (b) with the addition of PUDA powder. CNTs wrapped PUDA composite powder by electrostatic absorption were formed and precipitated from ethanol.

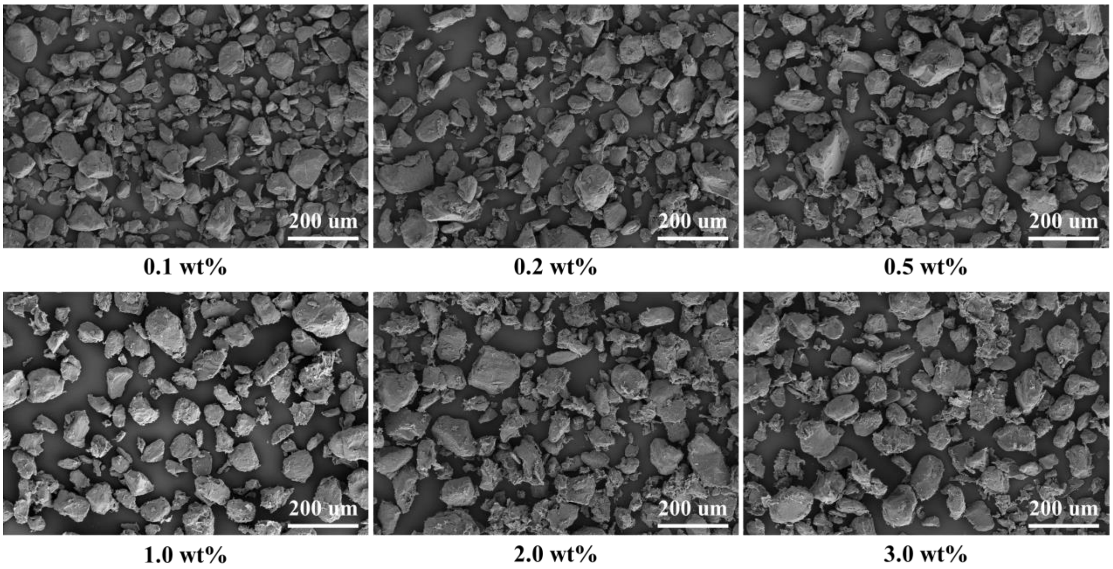

Figure S2. SEM images of CNTs wrapped PUDA composite powders with different CNTs loadings from 0.1 wt% to 3.0 wt%. There is no particles adhesion between CNTs wrapped PUDA powders.

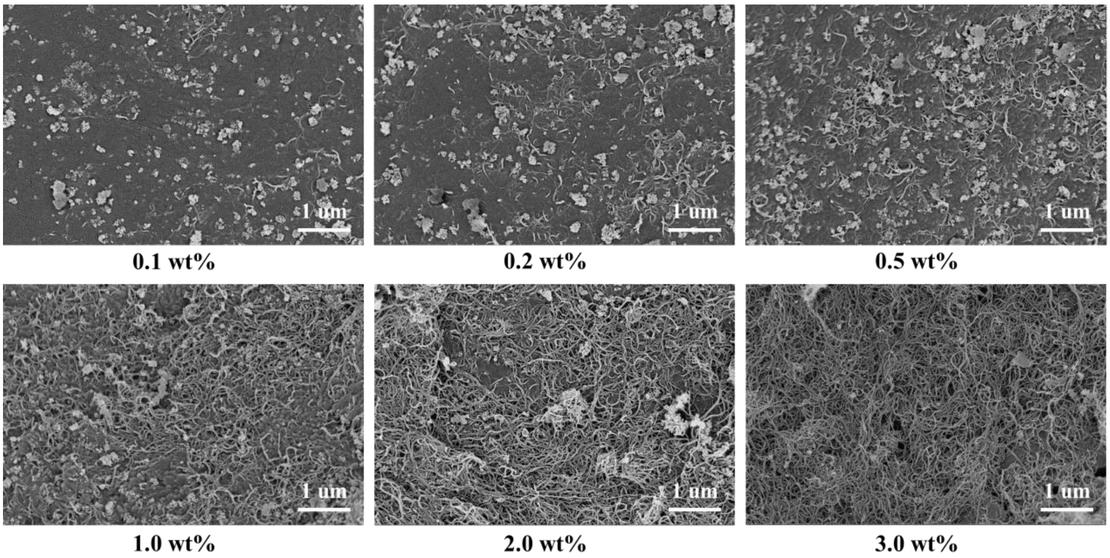

Figure S3. SEM images of the surface morphology of PUDA/CNTs composite powders with CNTs loading from 0.1 wt% to 3.0 wt%. The CNTs was uniformly dispersed on the surface of PUDA powder even at a high CNTs loading of 3.0 wt% and more CNTs was coated on the surface of PUDA powder with the increase of CNTs content.

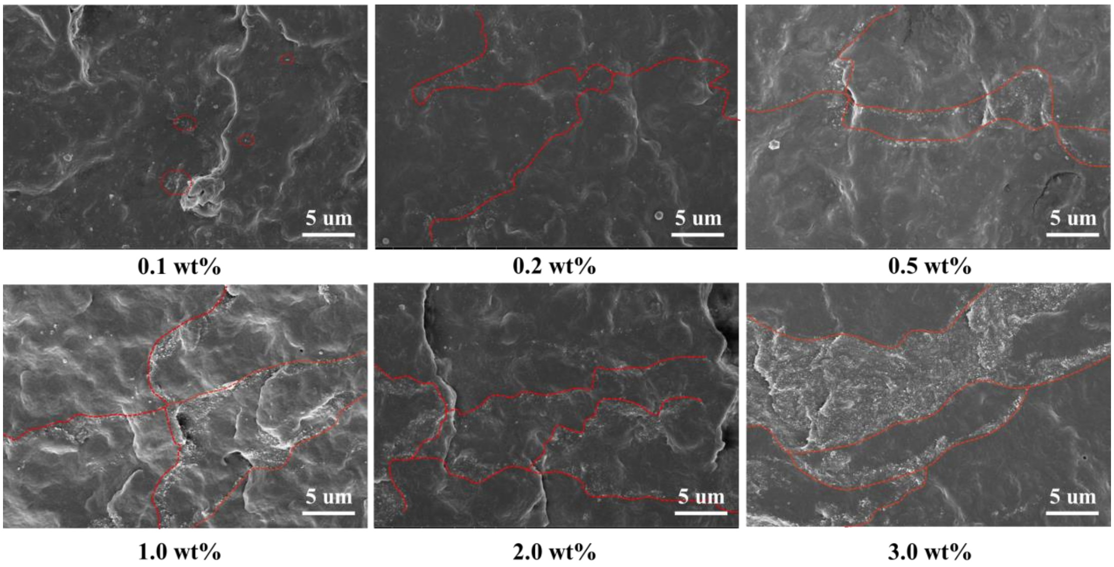

Figure S4. Cross-sectional SEM images of compression molded PUDA/CNTs composite prepared using CNTs wrapped PUDA composite powder with CNTs loading from 0.1 wt% to 3.0 wt%. Once the addition of CNTs is more than 0.2 wt%, obvious CNTs networks can be formed in the PUDA matrix, endowing high electrical conductivity properties to the composites. More complete CNTs networks were obtained with the increase of CNTs content.

Table S1. Tensile properties of compression molded PUDA/CNTs composites with different CNTs contents.

CNTs content (wt%)	Tensile strength (MPa)	Elongation at break (%)	Young's modulus (MPa)	Yield strength (MPa)
0	20.5 ± 0.9	1068 ± 14	257 ± 6	12.1 ± 0.4
0.1	28.7 ± 0.3	1210 ± 44	273 ± 3	13.8 ± 0.9
0.2	26.5 ± 0.2	1145 ± 32	293 ± 10	14.4 ± 3.1
0.5	26.2 ± 1.4	1124 ± 35	298 ± 29	14.9 ± 2.7
1	25.4 ± 0.5	1113 ± 61	323 ± 5	15.9 ± 2.5
2	21.9 ± 0.8	891 ± 149	435 ± 49	17.4 ± 1.4
3	19.3 ± 1.0	593 ± 86	444 ± 31	19.4 ± 1.2
10	13.5 ± 0.5	26 ± 2.5	539 ± 23	13.5 ± 0.5

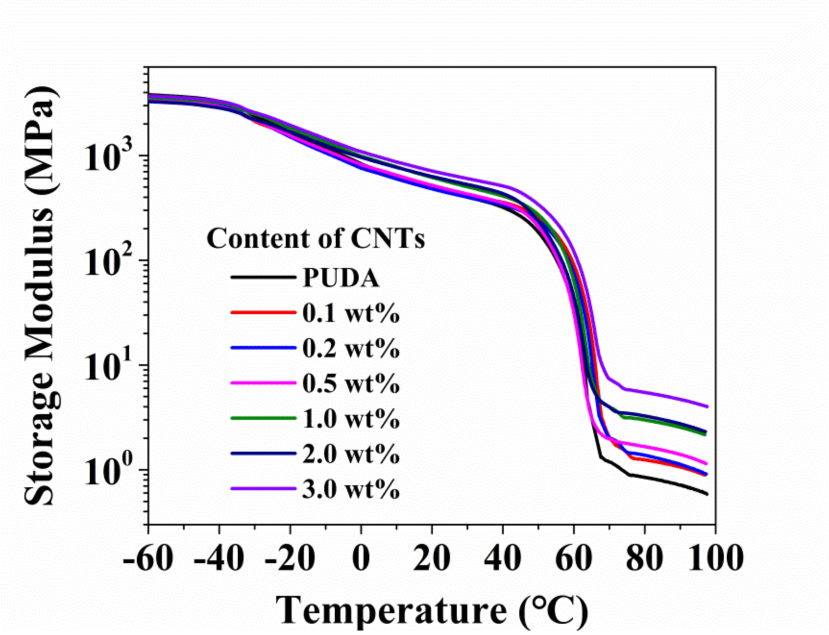

Figure S5. Storage modulus curves of PUDA/CNTs composites with different CNTs contents, suggesting a small effect of CNTs on the T_g of PUDA composite.

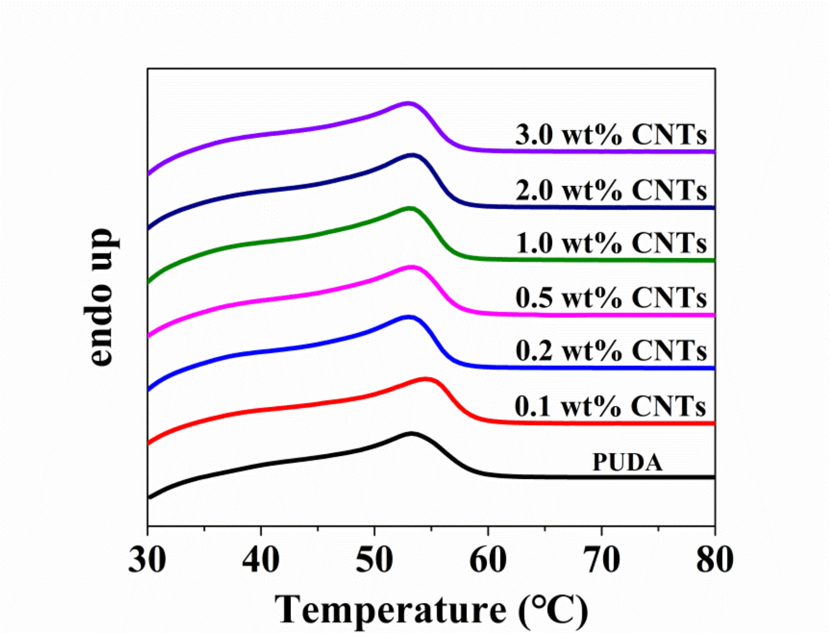

Figure S6. Differential scanning calorimetry (DSC) traces of PUDA/CNTs composites with different CNTs contents, showing the T_m of PCL segments in PUDA and the slight variation in T_m induced by the addition of CNTs.

To examine the electrothermal performance of PUDA/CNTs composites, the composite samples with a size of 45 mm × 10 mm × 0.85 mm were coated with silver paste on the two edges perpendicular to its long sides and connected to a direct current (DC) power supply. The time-dependent temperature changes of PUDA/CNTs composites under different applied voltages are shown in Figure S7 and the temperature can reach a plateau within 3 minutes. For PUDA/CNTs composites at the CNTs content above 1 wt%, a relatively high equilibrium temperature can be reach under a relatively low applied voltage due to the high electrical conductivity. The equilibrium temperature increases with the increase of applied voltage and the PUDA/CNTs composite with higher CNTs content shows better electrothermal capability. It is demonstrated that the conductive PUDA/CNTs composites show intensively electrothermal capability of efficiently transducing electrical energy into Joule heating energy. However, when the content of CNTs is lower than 0.5 wt%, the PUDA/CNTs composites show weak electrothermal performance due to the low conductivity.

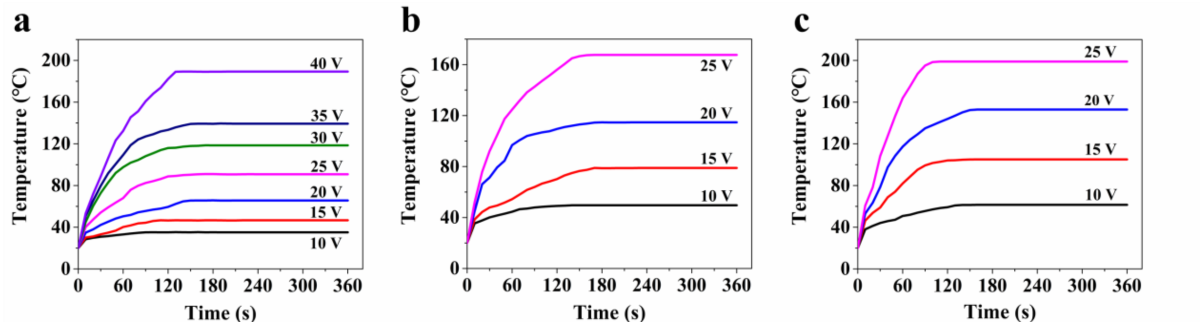

Figure S7. Temperature change as a function of time for PUDA/CNTs composites with different CNTs contents (a) 1.0 wt% CNTs, (b) 2.0 wt% CNTs and (c) 3.0 wt% CNTs upon applied varying voltages.

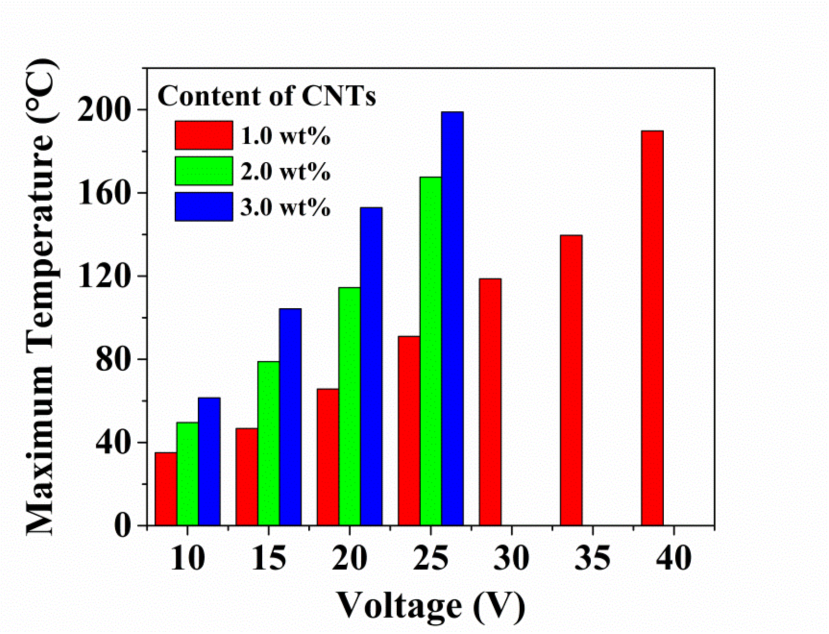

Figure S8. The maximum temperature can be obtained of PUDA/CNTs composites with different CNTs contents upon applied varying voltages.

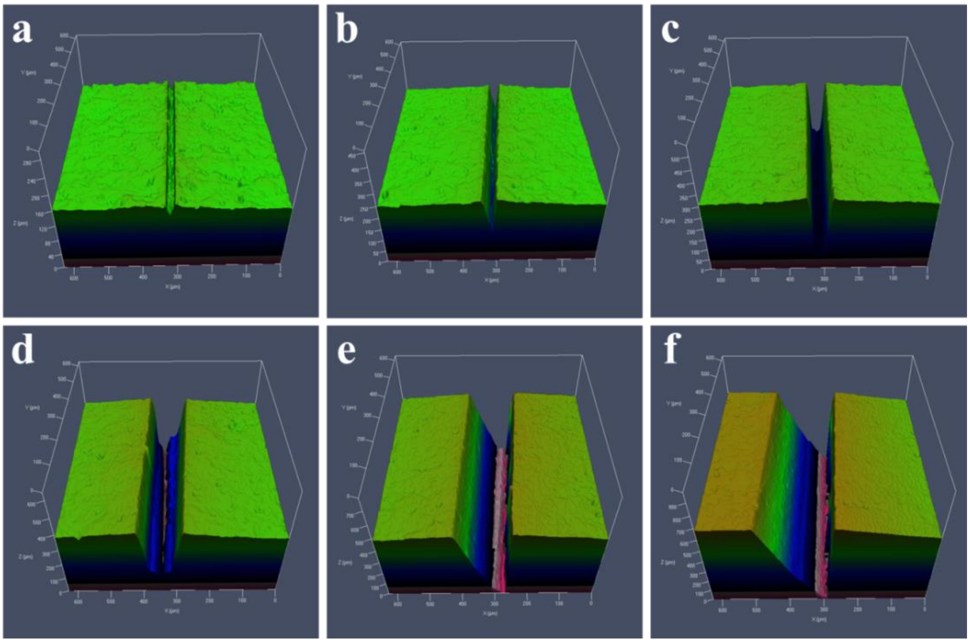

Figure S9. Laser Scanning Confocal Microscope (LSCM) images for the evolution of the crack with different depth. (a) $h_d \approx 51.2$ μm; (b) $h_d \approx 164.7$ μm; (c) $h_d \approx 340.6$ μm; (d) $h_d \approx 509.8$ μm; (e) $h_d \approx 643.4$ μm; (f) $h_d \approx 850$ μm. From the LSCM images, we can clearly measure the depth of the crack.

The remarkable mechanical strength and electrical conductivity self-healing performance make the PUDA/CNTs composites have potential applications in many fields. However, electric triggered repairing cannot be achieved when the damage breaks the electrical conductive property significantly and thus keeping electrical conductive is a premise. Therefore, we investigated the influence of crack depth on the resistance of the PUDA/CNTs composite at 1 wt% CNTs content. The composite sample (45 mm × 10 mm × 0.85 mm) was cut in the middle to form a crack pass along the entire length in a direction perpendicular to the current flow, and the depth was measured

using Laser Scanning Confocal Microscope (LSCM) (Figure S9). The resistance change of the sample induced by damaged crack was depicted as $\Delta R = R_d - R_0$, while the R_d is the resistance of damaged sample and R_0 is the initial resistance. We can depict the resistance change rate $\Delta R/R_0$ as the ratio between the resistance change ΔR and the initial resistance R_0. An increase trend in resistance change rate with crack depth change rate was observed (Figure S10). However, if there is just a scratch on the surface, a slight resistance increase appears. Interestingly, even under severe damage that the composite was cut completely with a little joint left, the resistance is only twice the original value (Table S2). The results demonstrate that our PUDA/CNTs composites can maintain their good electrical conductivity once damaged so as to achieve electricity-triggered self-healing process.

Table S2. The relationship between h_d/h_0 and $\Delta R/R_0$ of PUDA/CNTs composite with 1 wt% CNTs content.

Number	1	2	3	4	5	6
h_d / h_0 (%)	6	19.4	40.1	60	75.7	100
ΔR / R_0 (%)	1.1	2.3	5.9	13.8	30.8	98.4

h_d is the depth of the crack and h_0 is the thickness of the PUDA/CNTs sample.

$\Delta R = R_d - R_0$, R_d is the resistance of damaged sample and R_0 is the initial resistance.

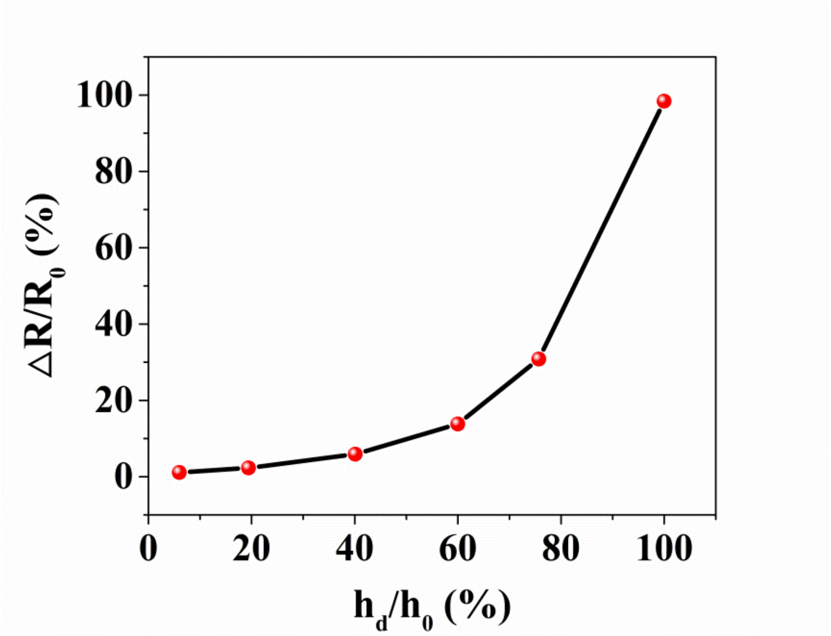

Figure S10. Resistance change rate as a function of depth change ratio of crack formed in PUDA/CNTs composite with 1 wt% CNTs content.

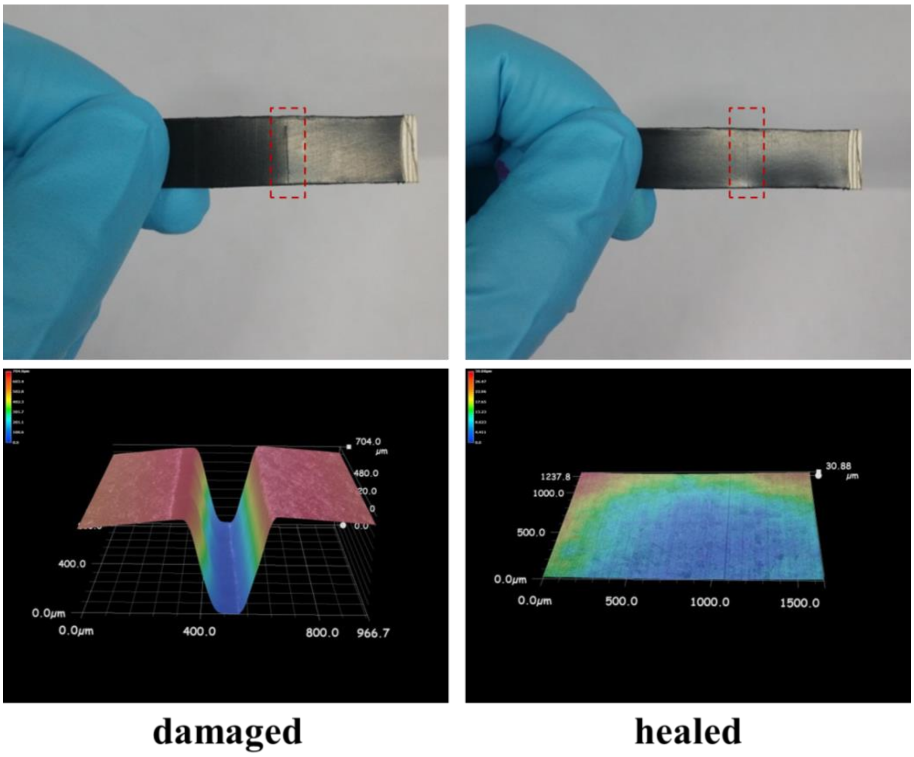

Figure S11. Photographs and 3D images of a damaged and healed PUDA/CNTs composite sheet, showing an obvious crack on the surface and the complete healing of the crack.

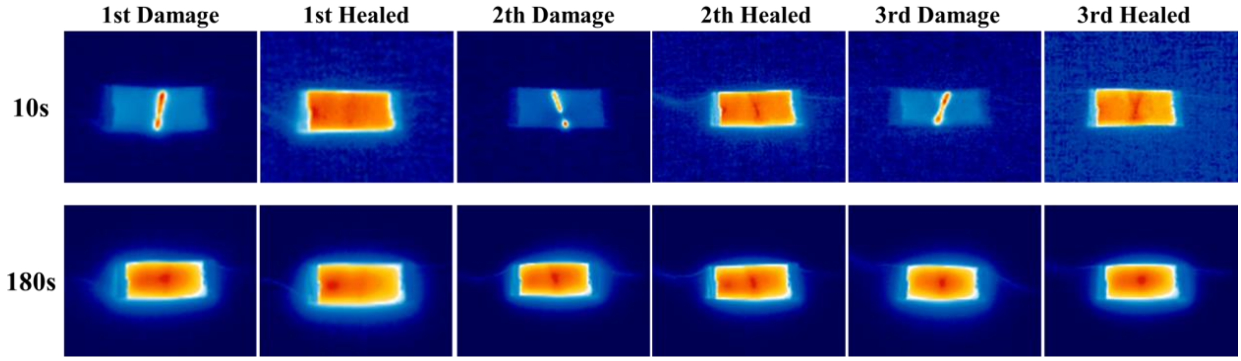

Figure S12. The thermal images of PUDA/CNTs composite sample at 1 wt% CNTs content with the size of 25 mm × 10 mm × 0.85 mm recorded by an Infrared Thermal Imager in three “damage-healing” cycles. The voltage of 20 V was applied.

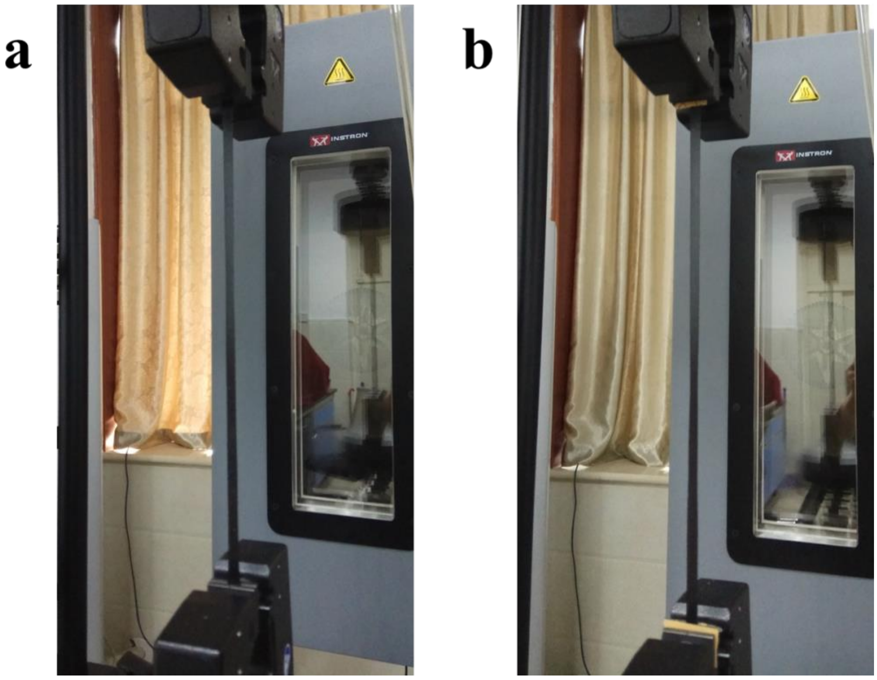

Figure S13. Photographs of the stretching of (a) original sample and (b) healed sample. (CNTs content: 1 wt%)

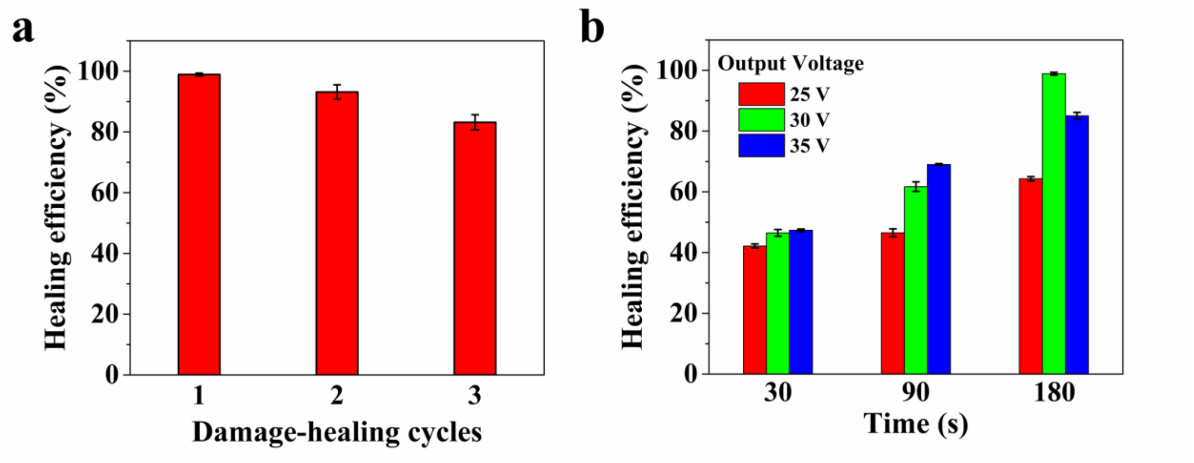

Figure S14. (a) Healing efficiency of PUDA/CNTs composite after multiple "damage-healing" cycles (CNTs content: 1 wt%, applied voltage: 30 V); (b) Healing efficiency of PUDA/CNTs composite induced by electricity at different treatment time (CNTs content: 1 wt%).

Normally the healing efficiency increases with increasing the voltage as the equilibrium temperature at low voltages cannot afford sufficient heating for retro-DA reaction in a short time. However a long time heating at high voltages may degrade the materials and leading to a low healing efficiency. The best healing efficiency for PUDA/CNTs composites at 1 wt% CNTs content can be obtained at a voltage of 30 V for 180 s.

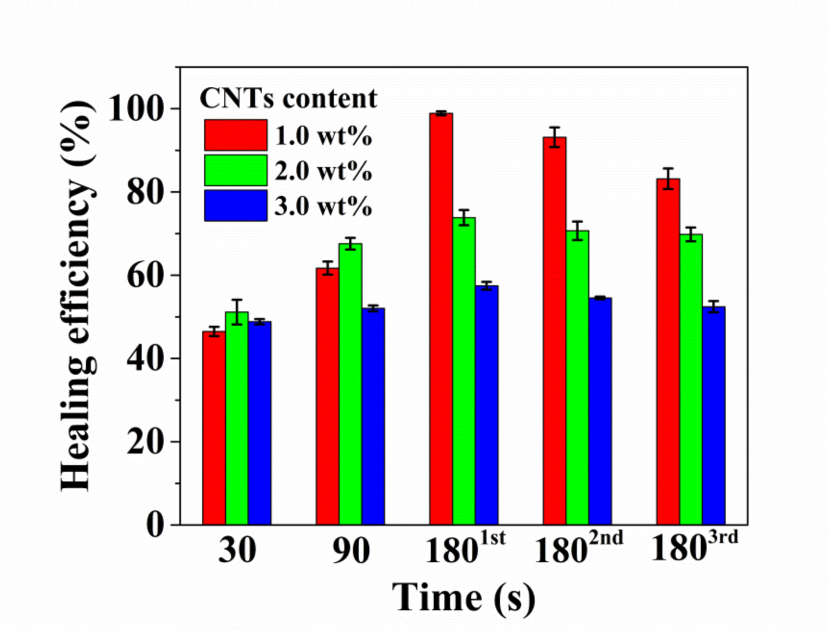

Figure S15. Healing efficiency of PUDA/CNTs composites with different CNTs content induced by optimal electricity at different treatment time and multiple "damage-healing" cycles (Applied voltage: 30 V for 1.0 wt% CNTs content; 25 V for 2.0 wt% CNTs content and 20 V for 3.0 wt% CNTs content).

The effect of CNTs contents on the healing efficiency of PUDA/CNTs was also investigated. Due to a stronger electrothermal effect at a higher CNTs loading, the PUDA/CNTs samples with 1, 2, and 3 wt% of CNTs content can obtain their best healing efficiencies by applying voltages of 30 V, 25 V and 20 V in 180 s, respectively. Normally the healing efficiencies increase with increasing CNTs contents. However, the healing efficiency in 180 s decreases when the CNTs content is above 1 wt%. This result can be explained that too many CNTs can hinder the retro-DA reaction and restrict the polymer chain mobility.

To examine the photothermal performance of PUDA/CNTs composites, the composite samples with a size of 10 mm × 10 mm × 0.85 mm were putting under the NIR light spot with 20 mm distance. The CNTs has good NIR absorbing and photothermal capacity, and thus PUDA/CNTs composites show strong NIR absorption and photothermal ability, which is totally different from the original NIR transparent PUDA matrix (Figure S16). The equilibrium temperature in the irradiated area can be obtained within 50 s under NIR light irradiation, suggesting the fast photothermal conversion. The maximum surface temperature of PUDA/CNTs composites is proportional to the NIR output power and exhibits a slight increase with the increase of CNTs contents. It can be explained that a little addition of CNTs even at a very low CNTs content of 0.1 wt% could induce almost saturated NIR absorption.

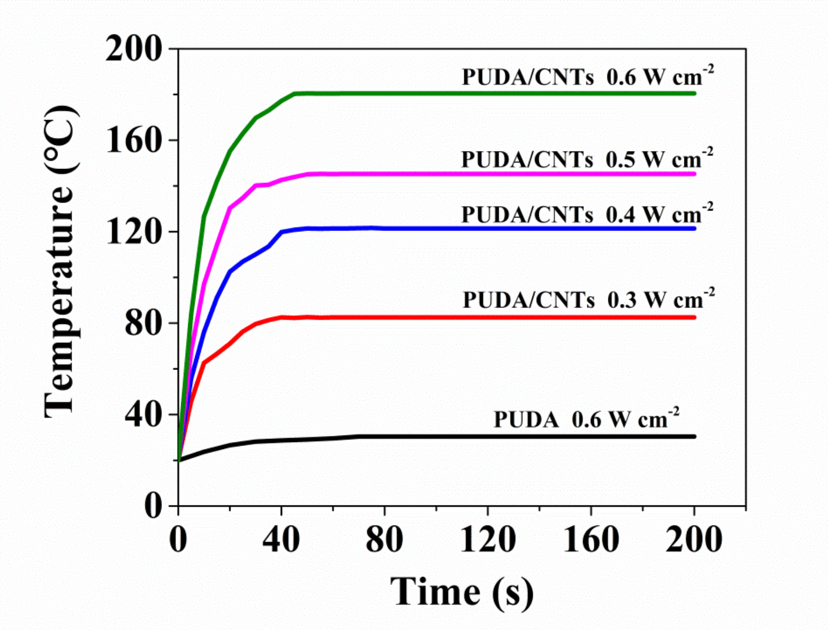

Figure S16. Temperature change as a function of irradiation time for PUDA/CNTs composite with 1.0 wt% CNTs content upon exposure to NIR light.

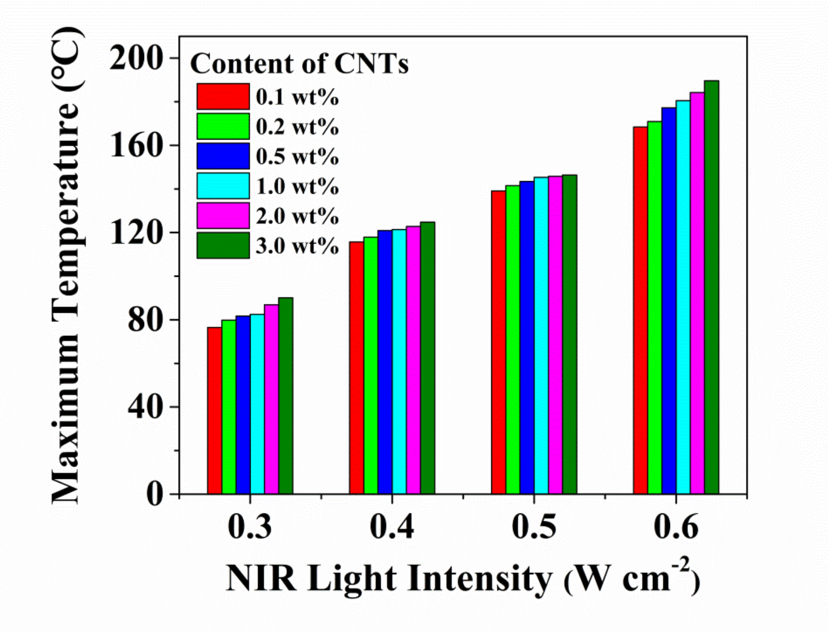

Figure S17. The maximum temperature can be obtained of PUDA/CNTs composites with different CNTs content upon exposure to NIR light with varying output power. (NIR treatment time: 180 s)

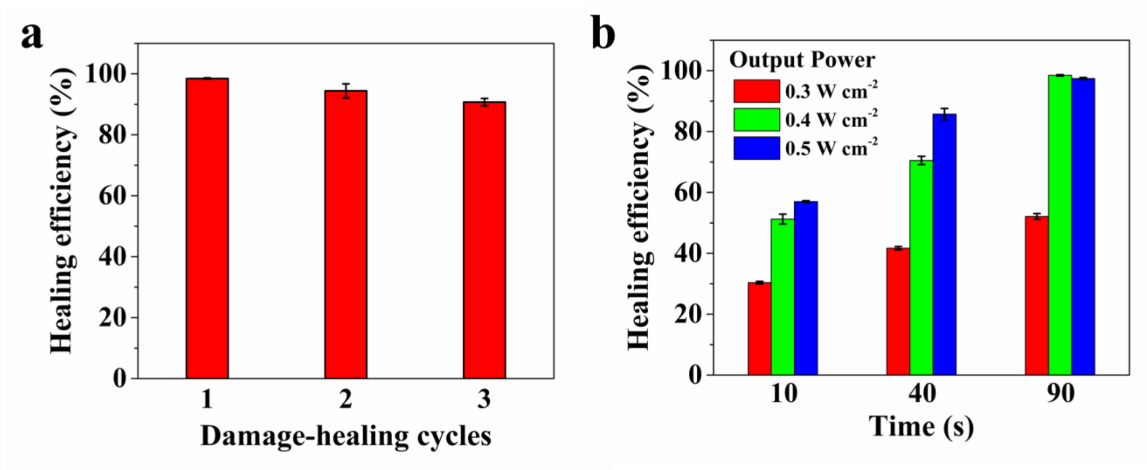

Figure S18. (a) Healing efficiency of PUDA/CNTs composite after multiple "damage-healing" cycles (CNTs content: 1 wt%, NIR light output power: 0.4 W cm^{-2}); (b) Healing efficiency of PUDA/CNTs composite induced by NIR light irradiation at different treatment time (CNTs content: 1 wt%). *The PUDA/CNTs composite exhibits excellent repeatable healing ability upon NIR light exposure and the healing efficiency after three "damage-healing" cycles still remains above 90%.*

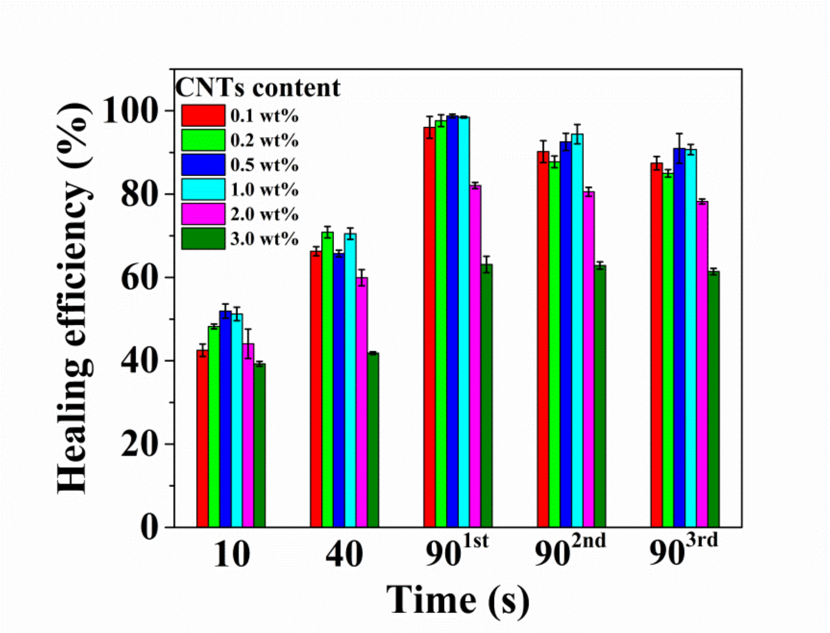

Figure S19. Healing efficiency of PUDA/CNTs composites with different CNTs content induced by optimal NIR light irradiation at different treatment time and multiple "damage-healing" cycles (NIR light output power: 0.4 W cm^{-2}).

When the CNTs content is between 0.1~1 wt%, the healing efficiency increases with the CNTs content in a short time but finally reaches the same for all samples in 90 s. When the CNTs content is higher than 2%, the healing efficiencies decrease. This is because a higher CNTs content will lead to a higher temperature which may degrade the materials and also too many CNTs will hamper the DA reaction.

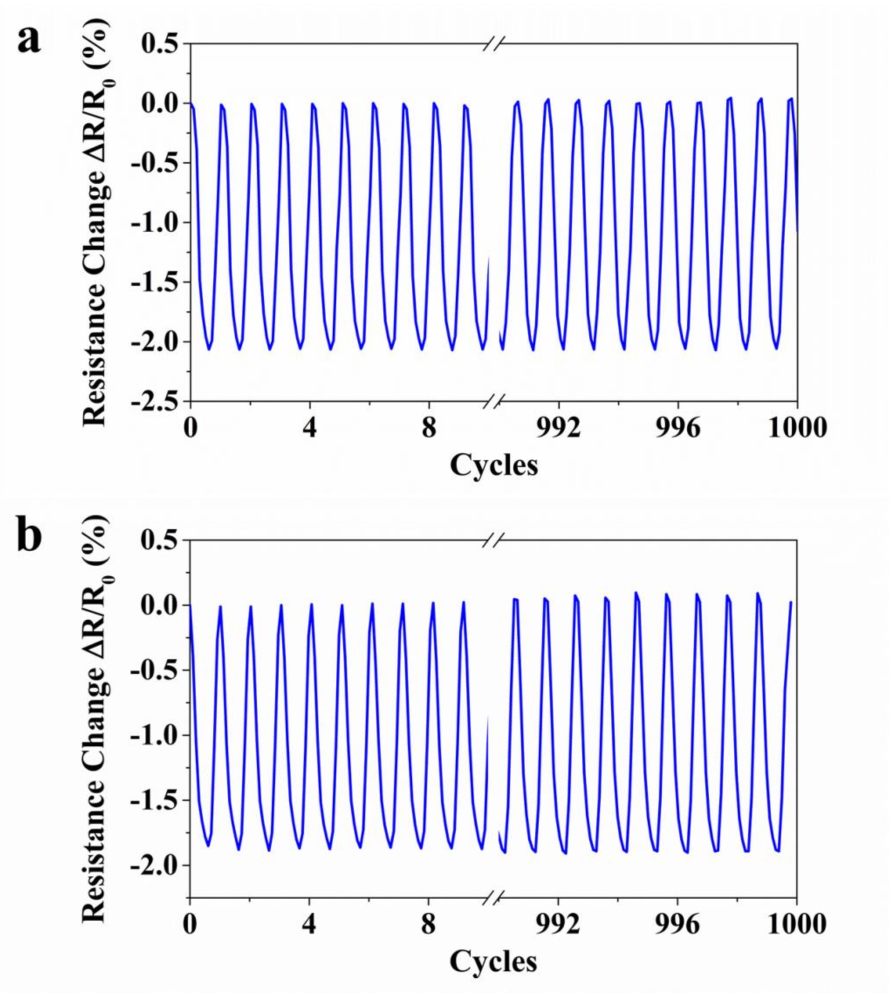

Figure S20. Cyclic bending tests on the PUDA/CNTs composite with 1 wt% CNTs content. (a) Resistance change of the original PUDA/CNTs composite during 1000 cycles of bending-straight at 0-20 % strain at a frequency of 3 cycles per second. (b) Resistance change of the self-healed PUDA/CNTs composite during 1000 cycles of bending-straight at 0-20 % strain at a frequency of 3 cycles per second.

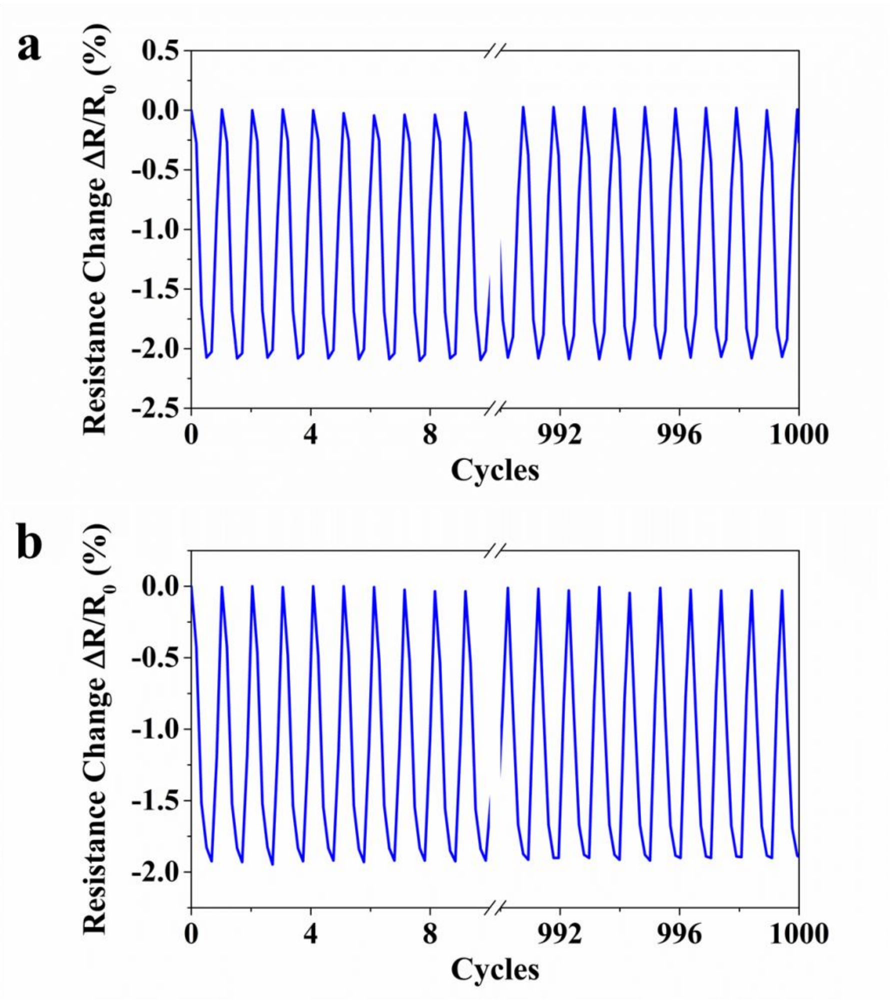

Figure S21. Cyclic bending tests on the PUDA/CNTs composite with 1 wt% CNTs content. (a) Resistance changes of the original PUDA/CNTs composite during 1000 cycles of bending-straight at 0-20 % strain at a higher frequency of 5 cycles per second. (b) Resistance changes of the self-healed PUDA/CNTs composite during 1000 cycles of bending-straight at 0-20 % strain at a higher frequency of 5 cycles per second.

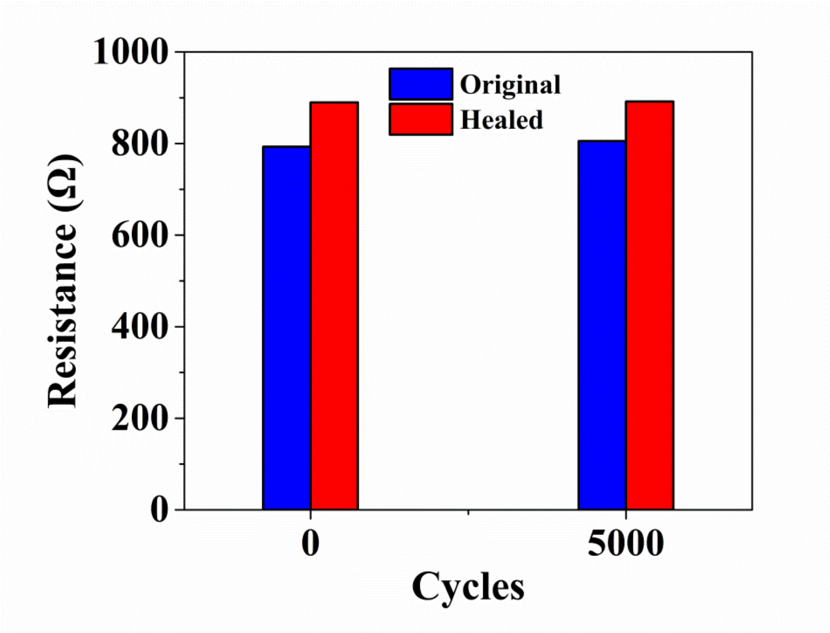

Figure S22. The resistance of the original PUDA/CNTs composite and self-healed PUDA/CNTs composite before and after 5000 cycles of bending-straight under 20 % applied strain. No obvious increase of resistance.
